# Characterization of Mechanical and Dielectric Properties of Silicone Rubber

**DOI:** 10.3390/polym13111831

**Published:** 2021-06-01

**Authors:** Eunyoung Cho, Loraine L. Y. Chiu, Mitchell Lee, Doshina Naila, Siddharth Sadanand, Stephen D. Waldman, Dafna Sussman

**Affiliations:** 1 Department of Electrical, Computer and Biomedical Engineering, Ryerson University, Toronto, ON M5B2K3, Canada; eunyoung.cho@ryerson.ca (E.C.); mitchell.lee@ryerson.ca (M.L.); dnaila@ryerson.ca (D.N.); ssadanand@ryerson.ca (S.S.); 2 Institute for Biomedical Engineering, Science and Technology (iBEST), Ryerson University and St. Michael’ Hospital, Toronto, ON M5B1T8, Canada; loraine.chiu@ryerson.ca (L.L.Y.C.); swaldman@ryerson.ca (S.D.W.); 3 Department of Chemical Engineering, Ryerson University, Toronto, ON M5B2K3, Canada; 4 Keenan Research Centre for Biomedical Science, The Li Ka Shing Knowledge Institute, St. Michael’s Hospital, Toronto, ON M5B1T8, Canada; 5 Department of Biomedical Physics, Ryerson University, Toronto, ON M5B2K3, Canada; 6 Department of Obstetrics and Gynaecology, University of Toronto, Toronto, ON M5G 1E2, Canada

**Keywords:** silicone rubber, dielectric properties, mechanical properties, dielectric constant, conductivity, compressive modulus, shear modulus

## Abstract

Silicone rubber’s silicone-oxygen backbones give unique material properties which are applicable in various biomedical devices. Due to the diversity of potential silicone rubber compositions, the material properties can vary widely. This paper characterizes the dielectric and mechanical properties of two different silicone rubbers, each with a different cure system, and in combination with silicone additives. A tactile mutator (Slacker™) and/or silicone thickener (Thi-vex™) were mixed with platinum-cured and condensation-cured silicone rubber in various concentrations. The dielectric constants, conductivities, and compressive and shear moduli were measured for each sample. Our study contributes novel information about the dielectric and mechanical properties of these two types of silicone rubber and how they change with the addition of two common silicone additives.

## 1. Introduction

Silicone rubber is a synthetic polymer consisting of silicone-oxygen backbones, which give unique material properties. These material properties include biocompatibility, superior temperature and chemical resistance, and good mechanical and electrical properties [1,2]. Due to its material properties, silicone rubber has a variety of applications in biomedical devices [1,3,4,5].

One of the applications for which silicone rubber is commonly used is in medical implantable devices, such as defibrillators, heart pumps, and surgical reconstructive components [3,6,7,8], as well as rehabilitation devices such as soft robotic systems [9,10,11].

Due to the diversity of potential silicone rubber compositions, the mechanical properties can vary widely. For example, the composition could be altered to make it exist in a rigid solid form or even in the form of a soft gel [2]. The mechanical properties of silicone rubber can, therefore, be manipulated to be tailored to an application. The choice of silicone rubber depends on the organ or site of interest, as well as the motion type and magnitude that the silicone implant could experience. Taking these factors into consideration ensures the durability and longevity of the implant.

Each organ and/or organ system has its own set of unique characteristics that must be upheld to deem the medical device or implant appropriate. Short-term implantable silicone, for example, is expected to function for a duration of up to 29 days [12]. These devices are often designed for contact with skin, bodily fluids, bone and tissue, electrosurgical devices, catheters, and even diagnostic guide wires. In a study conducted to determine the efficacy between two different silicone-mixed catheters, it was found that silicone, an inert material, can be combined with various noble metals and anti-bacterial compounds to decrease the incidence of infection in patients [13]. Congruently, silicone-based adhesives were studied for their biocompatibility and tested for their mechanical, adhesive, and biological properties. Here, different mixtures of silicone allowed for the fine tuning of the shear modulus, pull-off stress, adhesion energy, and stretch of films across rough and smooth surfaces, working towards the use of these products as potential adhesives in medical and healthcare-grade products [14].

Conversely, long-term implantable silicones are constructed to withstand more than 29 days in the human body, and often even until end of the patient’s life. These materials are biocompatible and must also uphold the physiological properties of the cells or organs that they are meant to support. Some examples of this grade of silicone which have been implanted in the body include those related to cardiovascular, knuckle, ocular, and toe implants, as well as defibrillators, heart pumps, and reconstructive components for surgery. Akin to other prosthetic devices, silicone implants must also be able to operate in synergy with communicative cells to maintain function. Using the example of medical implants, silicone has been successfully used in retinal prosthetics [15] and demonstrated its versatility in medicine. The retinal prosthesis was developed to improve high acuity vision over a larger area. In yet another example, silicone nanomembranes were developed for the treatment of heart-rhythm disorders and integrated electronics. In this study, the use of silicone allowed for the capacitive coupling between tissues, by acting as both a dielectric and as a robust, biocompatible barrier to prevent the penetration of biofluids into the electronics, thereby limiting the adverse reactions associated with this.

In each of the above applications, the ultimate choice of silicone rubber can also depend on the function of the silicone implants. For example, implants that undergo a mechanical motion need to be designed to sustain different types of force and torque. Another example is of implants inside or adjacent to primary organs or organ systems. In those cases, clinicians may opt to select a silicone material that is compatible with the diagnostic imaging modality that is most frequently used on that organ, to ensure medical diagnosis is not hindered in the future. In that case, a specific silicone may be chosen in order to result in the least amount of imaging artifacts, so as not to disrupt any future diagnosis. As such, it is important to characterize both the mechanical and dielectric properties of silicones to ensure the mechanical durability and imaging modality compatibility of the implants.

Low dielectric losses or low electrical conductivity is an important property of materials for medical implants. The dielectric losses contribute to continuous heat generation in the material, which can lead to dielectric breakdown, altering the necessary char characteristics for the silicone rubber to function appropriately [16].

The development of micro/nanofabrication technologies that can engineer diverse materials, such as silicone, has enabled the creation of novel types of bioelectronics for health monitoring and disease diagnostics [17]. The versatility of silicone, its malleability, manipulability, and biocompatibility, all contribute to the vast capabilities possible in medical research and in numerous applications not yet explored [18].

We investigated the dielectric properties and mechanical moduli of two silicone rubbers, each with a different curing system, and in combination with silicone additives. Specifically, we used platinum-cured and condensation-cured silicone, in combination with Thi-vex and additives, to comprehensively characterize the changes in dielectric and mechanical properties of silicone rubbers in relation to these additives. We hypothesized that the concentrations of the additives would correlate with the dielectric properties and mechanical moduli.

## 2. Materials and Methods

### 2.1. Sample Preparation for Dielectric Measurements of Silicone Rubber

In total, 40 samples were made: 36 of platinum-cured silicone (Dragon Skin 30, Smooth-on, Easton, PA, USA) and 4 of condensation-cured silicone (Mold Max XLS II, Smooth-on, Easton, PA, USA). The platinum-cured silicone was mixed at volume ratios of 1 to 0, 0.25, 0.50, 0.75, 1, 1.25, 1.50, 1.75, and 2, and subsequently with Thi-vex solution at concentrations of 0 *v/v*%, 0.5 *v/v*%, 1 *v/v*%, and 2 *v/v*% of the platinum-cured silicone component A. The condensation-cured silicone samples were made in duplicates by mixing the silicone with the Thi-vex solution in concentrations of 0%, 0.5%, 1%, and 2% of the condensation-cured silicone component A. The mixing of silicone solution and silicone additives was done by hand.

Each liquid sample was poured into 12-well cell culture plates. All samples were degassed using the periodic degassing method with a vacuum desiccator and a vacuum pump assembly for a fast and efficient degassing [19]. Then, the silicones were cured at room temperature for 16–24 h [20,21].

A Keysight N5232B PNA-L Network Analyzer (Keysight, Santa Rosa, CA, USA) with an N1501A high-temperature probe (Keysight, Santa Rosa, CA, USA) was used for measuring the dielectric properties of the samples in the range of frequencies 1–201 Mz. The frequency range covers the resonant frequencies of hydrogen atoms in 1.5T and 3.0T MRI scanners [22]. The presented dielectric constant and conductivity data in Table 1 were measured at 127 MHz, which is the resonant frequency of hydrogen atoms in a 3.0T MRI scanner. Keysight material measurement suite 2018 output the ε’ and ε” values with 4 decimal places in excel sheets. The conductivities of each sample were then calculated using the following equation,

σ = ε_0_ × ω × ε’ × tan δ (1)

where ω = γB, γ = 42.57747 × 10^6^ rad s^−1^·T^−1^, B = 3.0 T, ε_0_ = 8.85 × 10^−12^ F/m, and tan δ = ε”/ε’. The conductivity can be calculated by multiplying ε” in the data table and 0.00113, which is the approximate value of constants ω and ε_0_ multiplied. For calculating the error of measurement, we retrieved the following measurement accuracy information from the Keysight technical document [23].

ε’ = ε’ ± 0.05|ε*|(2)

ε” = ε” ± 0.05|ε*|(3)

Assuming that there is no error from ε_0_ and ω, we have the following equation for the error calculation [24].

Q = Ax(4)

where Q is the quantity being calculated, A is the exact parameter, and x has some error from measurement. Applying this to the conductivity equation, Q = σ, A = ε_0_ω and x = ε”. Taking the partial derivative with respect to x yields:

(5) $$\partial \sigma =\frac{\left|\sigma \right|}{\left|\varepsilon_{0}\omega \right|}\partial \varepsilon ”$$

With the differential parameters being the uncertainties, the error of the conductivity can be found. Additionally, it can be simplified to a normal derivative, since the differential equation is dependent only on x.

(6) $$d\sigma =\frac{\left|\sigma \right|}{\left|\varepsilon_{0}\omega \right|}d\varepsilon ”$$

The calculated error of conductivity ranged between 0.68–2.75%.

### 2.2. Sample Preparation for Compression and Shear Tests

Samples for compression and shear tests were prepared by curing silicone in in-house CAD-designed molds. The molds were of a cylindrical shape printed using a Form 2 resin printer (formlabs, Somerville, MA, USA). Platinum-cured silicone, condensation-cured silicone, and a Slacker additive (Smooth-on, Easton, PA, USA) were used in creating the samples.

Five duplicate samples were made for each of the silicone:Slacker ratios and for each mechanical test. The platinum-cured silicone was mixed at ratios of 1 to 0, 0.25, 0.50, 0.75, 1, and 1.25 of Slacker solution. As the elasticity of the silicone sample decreased dramatically at silicone:Slacker ratios beyond 1:1.25, while the sample stickiness increased, it was impossible to remove the sample from the mold without creating defects in their shape. Each liquid solution was poured into the printed mold and was degassed using the periodic degassing method with a vacuum desiccator and vacuum pump assembly for a fast and efficient degassing [19].

The technical data, which are provided by the manufacturer, indicate that the product is intended and designed for changing the tactile properties of platinum-cured silicone. The condensation-cured silicone is not one of the products with which the Slacker additive is intended to be mixed; thus, the condensation-cured silicone samples were not prepared with the Slacker additive.

The thickness and diameter of each sample was measured using digital calipers. Equilibrium mechanical properties of the samples in both compression and shear were then assessed using a two-axis (compression–shear) Mach-1 Micromechanical Testing System (Biomomentum, Laval, PQ, Canada) equipped with a si*x*-axis load cell, as described previously.

For the compression test, samples were first pre-loaded (5 mN), which was defined as the zero-strain state. Samples were then subjected to sequential step, uniaxial, unconfined compressions of 2% strain to a maximum of 10% strain (total of five steps). At each step, the resulting compressive force was recorded (at 10 Hz) until equilibrium was reached (force decay < 2 mN/min). The equilibrium compressive stress was calculated as the equilibrium compressive force normalized by the cross-sectional area of the sample and was plotted as a function of the applied strain. The equilibrium compression modulus was then determined from a numerical derivative of the equilibrium stress–strain curve at each step strain.

The same approach was used for shear testing, except that after pre-loading (5 mN compression), samples were then subjected to a sequential step, simple linear shears of 1% strain to a maximum of 5% strain. A total of *n* = 5 samples were tested for each experimental group, in both compression and shear.

### 2.3. Graph and Statistical Analysis

**Figure 1:**
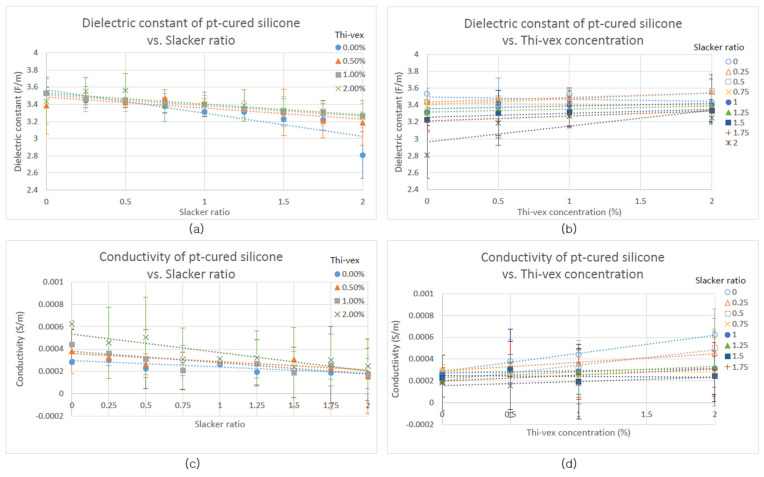
Dielectric properties of platinum-cured silicone samples relative to Slacker and Thi-vex concentrations. (**a**) Dielectric constant of platinum-cured silicone with various Thi-vex concentrations relative to Slacker ratio. (**b**) Dielectric constant of platinum-cured silicone at various Slacker ratios relative to Thi-vex concentrations. (**c**) Conductivity of platinum-cured silicone with various Thi-vex concentrations relative to Slacker ratio. (**d**) Conductivity of platinum-cured silicone at various silicone:Slacker ratios relative to Thi-vex concentration.

**Figure 2:**
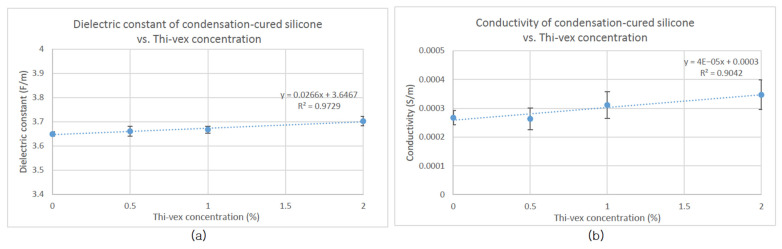
(**a**) Dielectric constant and (**b**) conductivity of condensation-cured silicone relative to Thi-vex concentration.

**Figure 3:**
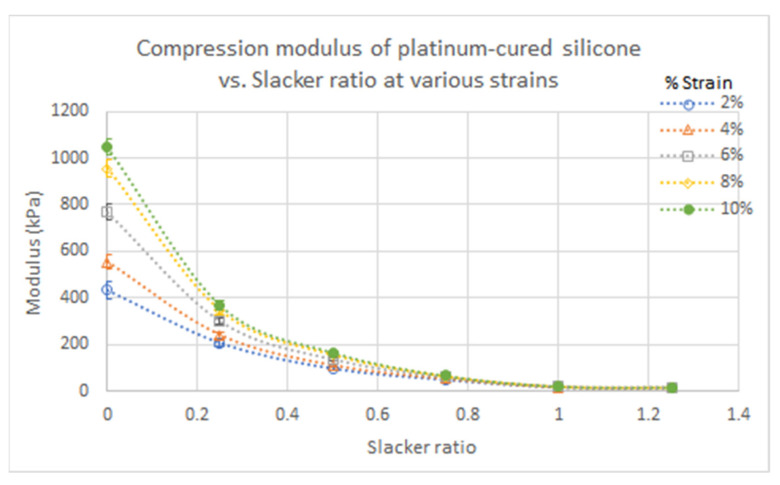
Compressive moduli of platinum-cured silicone at various % strains relative to silicone:Slacker ratio.

**Figure 4:**
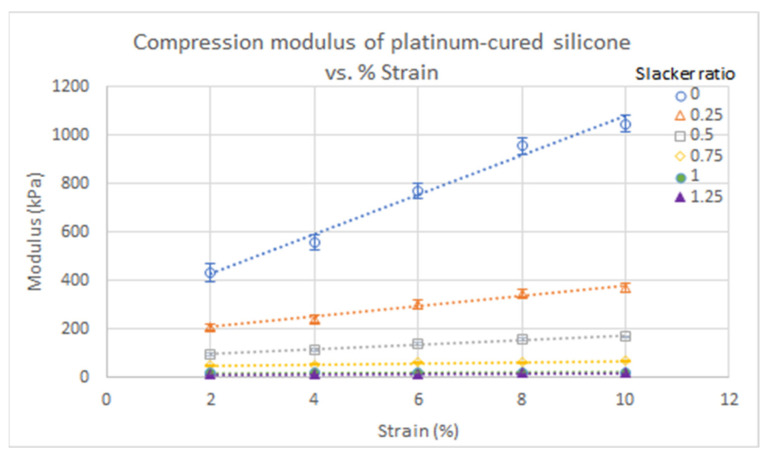
Compressive moduli of platinum-cured silicone at various silicone:Slacker ratios relative to % strain.

**Figure 5:**
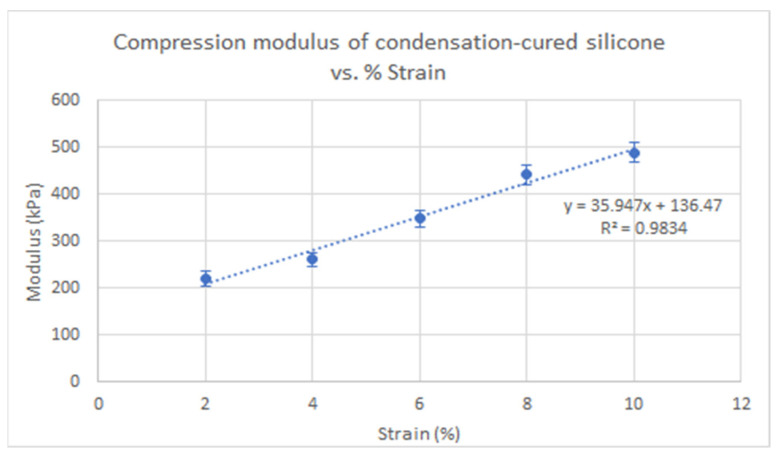
Compressive moduli of condensation-cured silicone rubber relative to % strain.

**Figure 6:**
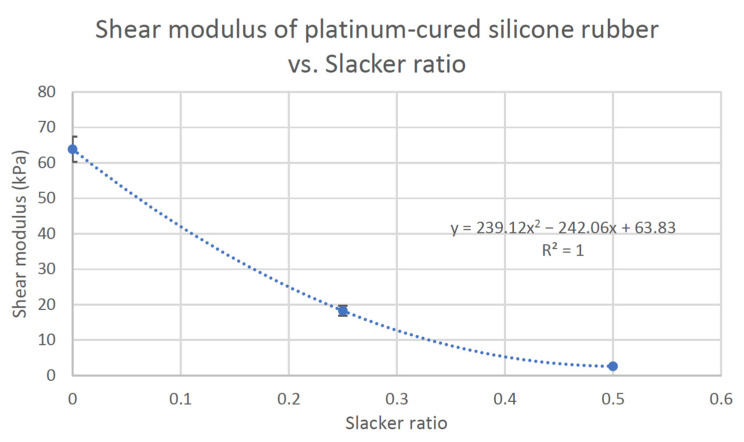
Shear moduli of platinum-cured silicone rubber relative to silicone:Slacker ratio.

Scatter plots of dielectric constant and conductivity values of each silicone sample were graphed as a function of Thi-vex concentration and Slacker ratio (Figure 1 and Figure 2) to determine how these affected the dielectric properties. Likewise, scatter plots of the compressive and shear moduli of the various types of silicone rubber were graphed as a function of Slacker concentration and percentage strain to determine how the mechanical strengths of the samples were affected by the Slacker concentration (Figure 3, Figure 4, Figure 5 and Figure 6). The plotted data are the average value of five measurements of each sample. A standard deviation was calculated for each average and are presented as error bars in Figure 1, Figure 2, Figure 3, Figure 4, Figure 5 and Figure 6.

To compare the goodness of fit, the R^2^ of the trendline in each figure was plotted with their respective function equations. In addition, the standard deviation of each grouped data was plotted as error bars in each figure (Figure 1, Figure 2, Figure 3, Figure 4, Figure 5 and Figure 6). The sample groups in each figure were compared by using a two-way ANOVA test to determine if there was a significant difference among the sample groups. A two-way ANOVA with post-hoc test was performed on a spreadsheet available on a webpage named AtoZmath [25], which was built for this specific test. In addition, we performed a two-sample t-test using Excel (Microsoft, Redmond, Washington) to determine which particular sample groups had a significant difference. Lastly, a regression slope test using Excel (Microsoft, Redmond, Washington) was employed for determining if a slope was significantly different from zero. *p* values smaller than 0.05 were considered significant.

## 3. Results

### 3.1. Results of Dielectric Measurements

For platinum-cured silicone rubber samples, the dielectric constant was found to range between 2.81 and 3.56 F/m, and the conductivity ranged between 0.000154 and 0.000622 S/m. For condensation-cured silicone rubber samples, the dielectric constant was found to range between 3.65 and 3.70 F/m, and the conductivity ranged between 0.000263 and 0.000347 S/m. These results are summarized in Table 1 and Table 2.

The dielectric constant of platinum-cured silicone was found to decrease as the silicone:Slacker ratio increased (R^2^ of each regression is in Table A1 Appendix A) and all decreasing linear relationships were found to be significant (*p* < 0.05). The regression slope test demonstrated that the dielectric constants of platinum-cured silicone at a 1:1.25 silicone:Slacker ratio had a significant increasing linear relationship with Thi-vex concentration (*p* < 0.05); however, at other silicone:Slacker ratios, no significant linear relationship was observed (R^2^ of each regression is included in Table A2 Appendix A).

The conductivity of platinum-cured silicone decreased as the silicone:Slacker ratio increased (R^2^ of each regression is included in Table A3 Appendix A), and all decreasing linear relationships were found to be significant (*p* < 0.05). The regression slope test demonstrated that the conductivity of platinum-cured silicone had a statistically significant increasing linear relationships with Thi-vex concentrations at 1:0.00, 1:0.25, 1:0.50, and 1:1.75 silicone:Slacker ratios (R^2^ of each regression is included in Table A4 Appendix A).

The 0.00% Thi-vex platinum-cured silicone sample group had the most rapidly decreasing dielectric constant rate relative to silicone:Slacker ratio, while the slopes of the other Thi-vex concentration groups had no significant differences among them (*p* > 0.05) and were parallel to each other, as shown in Figure 1a. The 1:2.00 silicone:Slacker ratio had the most rapidly increasing dielectric constant rate relative to Thi-vex concentration, as shown in Figure 1b.

As depicted in Figure 1c, the higher Thi-vex concentration sample group had a more rapidly decreasing conductivity rate than the lower Thi-vex concentration sample group. In addition, the lower silicone:Slacker ratio sample group mostly had a more rapidly increasing conductivity rate than the higher silicone:Slacker ratio sample group, as shown in Figure 1d.

The results of the two-way ANOVA tests demonstrated that there were significant differences between the sample groups of various silicone:Slacker ratios (*p* < 0.05) and the sample groups of various Thi-vex groups (*p* < 0.05) in Figure 1. A two-sample t-test was performed in order to determine which particular sample groups had significant differences. The results demonstrated that no significant difference in dielectric constant data was observed among various Thi-vex platinum-cured silicone sample groups shown in Figure 1a (*p* > 0.05). A significant difference in conductivity data between the 0% and 2% Thi-vex platinum-cured silicone sample groups is shown in Figure 1c (*p* < 0.05). Some significant differences in dielectric constant data, shown in Figure 1b, were observed between the 1:0.00 silicone:Slacker ratio sample group and the 1:1.75 and 1:2.00 silicone:Slacker sample groups; between the 1:0.25 silicone:Slacker ratio sample group and the 1:1.75 and 1:2.00 silicone:Slacker sample groups; between the 1:0.75 and 1:2.00 silicone:Slacker ratio groups; between the 1:1.00 and 1:2.00 silicone:Slacker ratio groups; and between the 1:1.25 and 1:2.00 silicone:Slacker ratio groups (*p* < 0.05). Some significant differences in conductivity data are shown in Figure 1d between the 1:0.00 silicone:Slacker sample group and the sample groups in which the silicone:Slacker ratio was higher than 1:1.50 (*p* < 0.05). Table A5, Table A6 and Table A7 Appendix A summarize whether there was a significant difference in dielectric properties between one concentration group and another.

Figure 2a shows a proportional relationship between the dielectric constant of condensation-cured silicone and Thi-vex concentration (R^2^ = 0.97). The conductivity of condensation-cured silicone also increased with increasing Thi-vex concentration, as shown in Figure 2b (R^2^ = 0.90). A regression slope test was performed on the slopes of both Figure 2a,b and demonstrated that the linear increasing relationships between the dielectric properties and Thi-vex concentration were significant (*p* < 0.05).

### 3.2. Results of Mechanical Testing

A compression test was performed on the platinum-cured silicone rubber with Slacker ratios of 1:0.00–1.25 and the condensation-cured silicone rubber without Slacker at 2–10% strain. The results are summarized in Table 3. Significant differences among sample groups with different % strain in, Figure 3, were observed by performing a two-way ANOVA with post hoc test. Similarly, the same statistical test was performed on sample groups with different slacker ratios, in Figure 4. Furthermore, a regression slope test was performed on the trendlines in Figure 4 and Figure 5 to determine if the increasing slope was significant.

A shear test was performed on the platinum-cured silicone rubber with Slacker ratios of 1:0.00–0.75 and the condensation-cured silicone rubber without Slacker. The platinum-cured silicone samples with a Slacker ratio greater than 1:0.75 did not produce observable data. These results are summarized in Table 4.

The compressive moduli of the platinum-cured silicone rubber decreased with increasing Slacker ratio at all % strains, as shown in Figure 3. The linear equations and R^2^ of each trendline are shown in Table A8 Appendix A. Statistical analysis revealed a decreasing exponential trend for compressive modulus as a function of Slacker ratio, as seen in the trendlines in Figure 3. As the Slacker ratio increased by 0.25, the compressive modulus generally decreased by ⅓ to ½.

The regression slope test demonstrated that the compressive moduli of the platinum-cured silicone rubber at all Slacker ratios increased with increasing % strain (Figure 4, *p* < 0.05). The platinum-cured silicone rubber with a smaller Slacker ratio recorded larger differences in moduli between % strains, as reflected by the slopes of the trendlines in Table A9 Appendix A. As shown in Figure 4, the difference in moduli between the samples with a lower Slacker ratio and the samples with a higher Slacker ratio became greater as the % strain increased. The differences between moduli became significantly smaller between 1:1.00 and 1:1.25 Slacker ratios at all % strains. At a 1:1.25 Slacker ratio, the smallest differences between compressive moduli at various % strains were observed. The result of two-way ANOVA demonstrated that there was no significant difference between the sample groups of various strains (*p* > 0.05) in Figure 3, but there was a significant difference between groups of various silicone:Slacker ratios (*p* < 0.05) in Figure 4. The results of the regression slope test demonstrated that all linear trendlines of the various silicone:Slacker ratios in Figure 4 had a significant increasing linear relationship between the compressive modulus and % strain (*p* < 0.05).

The compressive modulus of the condensation-cured silicone was found to have an increasing linear relationship with % strain (*p* < 0.05), as shown in Figure 5 (R^2^ = 0.98) by performing a regression slope test. The compressive moduli of the condensation-cured silicone were lower than those of pure platinum-cured silicone at corresponding strains. The compressive modulus of condensation-cured silicone at 2% strain was 219.75 kPa, which is close to the value of platinum-cured silicone at 1:0.25 Slacker ratio at 2% strain, 206.81 kPa. However, the increasing rate of compressive modulus was greater for the condensation-cured silicone in Figure 5 compared to that of the platinum-cured silicone at 1:0.25 Slacker ratio in Figure 4.

The measured shear moduli of the platinum-cured samples resulted in a second order polynomial decay curve in Figure 6 (R^2^ = 1). The polynomial fit was arrived upon by comparing the R^2^ values of various trend lines, including linear, exponential, log arithmetic, polynomial, and power, and selecting the one with the highest R^2^. The shear modulus of the condensation-cured silicone was higher (86.91 kPa) than that of the pure platinum-cured silicone (63.83 kPa).

## 4. Discussions

### 4.1. Dielectric Properties and Concentrations of Slacker Additive and Thi-Vex

The dielectric constant and conductivity of the platinum-cured silicone rubber decreased as the Slacker additive concentration increased, as shown in Figure 1a,c. This indicates that the molecular and structural change induced by the addition of Slacker is related to the dielectric constant and conductivity of the platinum-cured silicone rubber.

The platinum-cured silicone rubber consists of two parts of translucent viscous solution. The cross-linking molecules and platinum catalysts are found in different parts, A and B, and the mixing of the two solutions occurs in the cross-linking reaction.

The crosslinked network density results in a lower dielectric constant and lower dielectric loss by hindering molecular motion under an external electric field [26]. As the structure of a silicone polymer becomes oriented, the less electrical polarization and conductance will occur, and this decreases the dielectric constant and conductivity of the polymer sample.

While the conductivity of the silicone rubber may increase as cross-linking density decreases and there is a higher concentration of mobile electrons, Slacker additive itself might have a very low conductivity. According to the literature, the addition of long prepolymers results in a higher electrical conductivity [27]. The polymerization and cross-linking reaction caused by crosslinking molecules and platinum catalysts increases the length of polymer chains. The polymers in Slacker additive can be assumed to have chain-terminating groups which can bind to reactive side groups of silicone polymer molecules. As the Slacker ratio increases, mobile charges that would have otherwise been on reactive side groups of silicone rubber parts A and B are now bound in chain-terminating groups by Slacker. This results in the shortening of each polymer chain, which leads to a decrease in the conductivity of the platinum-cured silicone rubber.

The dielectric constant of silicone elastomer increases with the degree of polymerization of the siloxane backbone [28]. The dielectric constant is related to the siloxane-to-methyl group ratio, which quickly increases at the beginning of polymerization [29]. Since the addition of Slacker additive decreases the length of the polymer chains and the size of molecular networks by binding its chain-terminating groups with reactive side groups from silicone rubber parts A and B, the degree of polymerization of the siloxane backbone decreases with increasing Slacker ratio.

The saturation of the chain-terminating groups of Slacker additive decreases both the dielectric constant and conductivity. However, the effect of chain-terminating groups in decreasing the dielectric constant and conductivity may be offset by the effect of decreasing the cross-linking density, which increases the dielectric constant and conductivity. These offset effects explain the shallow increasing trend lines that are shown in Figure 1a,c.

Increasing the concentration of Thi-vex in either platinum-cured or condensation-cured silicone samples moderately increased both the dielectric constant and conductivity in several samples, as shown in Figure 1b,d. Thi-vex is a silicone thickener which increases the viscosity of a silicone solution. According to the literature, the viscosity of silicone elastomer can be increased in two ways: (1) increasing the chain length in a linear manner with the addition of Si-O units, or (2) increasing the chain length and employing a cross-linker [30]. Therefore, a silicone thickener can be considered to consist of Si-O units and/or cross-linking molecules of silicone rubber. However, Thi-vex is designed for various types of silicone rubber that employ different curing methods and different cross-linking molecules [31]. Therefore, Thi-vex is more likely to consist of Si-O units rather than cross-linking molecules, increasing the viscosity or the liquid’s resistance to flow by reinforcing the intermolecular forces of attraction within a liquid [26]. Intermolecular forces ultimately derive from the electrostatic properties of molecules. Therefore, as the intermolecular forces become greater within a sample, the dielectric constant and conductivity of a sample increase.

### 4.2. Compressive/Shear Moduli of Various Silicone Rubber

As shown in Figure 3 and Figure 6, both compressive and shear moduli decreased with increasing Slacker ratio in platinum-cured silicone samples, due to the effect of the cross-link density [32]. As the Slacker additive ratio increases in the silicone samples, the density of cross-links decreases and this makes the silicone samples less stiff and less rigid, resulting in lower compressive and shear moduli. Similarly, less stiff silicone samples require less external force to produce deformation, resulting in smaller slopes relating compressive modulus to % strain for higher Slacker ratios in Figure 6. On the other hand, pure, or lower Slacker-ratio silicone samples contain abundant cross-links, resulting in greater elasticity in the samples and an increased ability to resist deformation.

Slacker is a commercial silicone additive which is designed for changing the tactile properties of platinum-cured silicone rubber [33]. The cross-linking reaction forms bridges across linear polymer chains to create large, branched molecules. During the formation of cross-links, the mixed silicone solution loses its fluid properties and gains rigidity as a solid material [34]. However, with the increasing Slacker additive ratio in the silicone samples, the samples became less rigid and more of a gel-like material. Observing the change in property of the material, it can be assumed that the order of cross-linking is reduced due to the addition of Slacker additive. Increasing slacker additive ratio decreases the volume of silicone parts A and B in a sample. Hence, increasing Slacker additive decreases the number of cross-linking molecules and platinum catalyst in the mixed sample solution, which leads to a decrease in the cross-linking density.

A study investigated the effect of the amount of Slacker additive on the material properties of silicone rubbers. Stoll et al. demonstrated that an increasing content of Slacker additive increased the storage modulus of silicone rubber [35]. For a cross-linked polymer, the storage modulus value in the rubbery plateau region is inversely correlated with the number of cross-links in the polymer [36,37,38]. The polymer cross-linking density can be quantitatively calculated by using the measured storage modulus [37,38]. Although our study did not collect the storage modulus of samples, the relationship between the addition of Slacker additive and the decrease in cross-linking density was proven.

From the results, the platinum-cured silicone rubber was found to have a higher compressive modulus, while it had a lower shear modulus compared to the condensation-cured silicone rubber. This implies the platinum-cured and condensation-cured silicone rubbers have different molecular structures; while one structure makes the silicone rubber resistant to compression but easier to shear, the other structure results in the opposite properties.

A dense 3D network or high cross-link density is a factor that increases the shear modulus of a material. Silicone rubber is a synthetic polymer which undergoes a cross-linking reaction that solidifies it. The cross-linking reaction forms covalent bonds that bridge across polymer chains to create a large, branched molecule network in three dimensions. Platinum-cured silicone rubber uses an additional curing method that functions by attaching Si-H groups in a crosslinker to vinyl functional groups (C=C) in other polymer chains [1,39]. The crosslinking of condensation-cured silicone rubber is based on the reaction between hydrolysable Si-X groups in crosslinkers and Si-OH groups in other polymer chains [1,40]. The crosslinking molecule used in platinum-cured silicone rubber is polymethylhydrosiloxane, a long polymer chain that consists of a repeated silicon and oxygen backbone, and functional groups and hydrogen that are bonded to silicon atoms of the backbone [1]. Silicic acid is generally used in the condensation-cured silicone rubber as the crosslinking molecule [40]. According to the literature, only the polymer ends with Si-H groups of polymethylhydrosiloxane, the crosslinking molecule of the platinum-cured silicone rubber is reactive, whereas all four ends of silicic acid, the crosslinking molecule of condensation-cured silicone rubber, are Si-OH groups which are reactive [1,39,40].

Thus, the crosslinker of condensation-cured silicone rubber leads to the formation of a denser 3D network of crosslinked polymers. The increased crosslinking density decreases with the specific volume of the polymer. For example, the specific volume of the platinum-cured silicone rubber is 25.7 in^3^/lb (92 × 10^−5^ m^3^/kg) [20], whereas the specific volume of the condensation-cured silicone rubber is 22.7 in^3^/lb (82 × 10^−5^ m^3^/kg) [21]. With increasing cross-link density, the movement of molecules involved in cross-links is restricted because the distortion and stretch of the chemical bonds are limited. Therefore, a denser 3D network of crosslinked polymers in the condensation-cured silicone rubber resulted in a higher shear modulus.

As the density of the 3D network increases, the compressive modulus may increase because the free volume in the material decreases, allowing smaller volumes of the molecules to contract. However, we found that the condensation-cured silicone rubber with a denser 3D network had a lower compressive modulus than the platinum-cured silicone rubber. We hypothesize that this is due to the high compressive modulus of the platinum-cured silicone rubber, which is a result of the abundant C-C bonds. A weak bond directionality is a factor that leads to greater transverse strain and less longitudinal strain in a material [41]. A nonpolar covalent bond that has an equal distribution of electron density, has a lower bond directionality compared to a polar covalent bond.

Furthermore, a nonpolar covalent bond is more resistant to compression, which shortens the interatomic distance relative to a polar covalent or ionic bond [42]. The C-C bond, a nonpolar covalent bond, is abundant in platinum-cured silicone rubber, whereas it is less abundant in condensation-cured silicone rubber [1,39,40]. Thus, the platinum-cured silicone rubber has an overall weaker bond directionality, causing a lower shear modulus and a higher compressive modulus compared to those of condensation-cured silicone rubber.

In conclusion, the differences between the compressive and shear moduli of platinum-cured and condensation-cured silicone rubber are due to the differences in the packing density of their polymer chains and their bond directionality. In this study, we measured the compressive and shear moduli of two silicone rubbers undergoing 2–10% strains and 1–5% strains, respectively. Based on the function of the medical device and location where the device is placed or implanted, some applications would require more extensive testing with a higher percentage strain. However, the selected values were sufficient for our purposes. 

While silicones should have a Poisson’s ratio of 0.5 and the relation of E = G × (1 + 2*nu) or 2G should be fulfilled, it is not immediately apparent in our figures and tables that the reported elastic and shear moduli fulfilled this requirement. This is because of our testing methods. For compression, the ratio of resulting stress to the applied strain (compressive elastic modulus) is constant at very low strains, but this linearity ceases as the strain increases. This is evident in the increasing compressive elastic modulus with increasing strain, as reported in Table 3 and Figure 4 and Figure 5; and this is related to the bulging of the free sides (and, therefore, increase in area) during compression. For shear, the linearity remains to much higher strains, as there is no change in area throughout the test, so the shear modulus is constant with increasing strains. As such, only one value for shear modulus was reported for each sample across all tested % strains. The relation of E = 2G might be fulfilled at lower strains, at which the compressive elastic modulus would be much lower.

### 4.3. Comparison with Other Literature Values

According to the technical documents of various commercial silicone rubbers, the typical values of dielectric constant are in the range of 2.6–3.4 F/m [21,43,44,45]. According to other literature, the range of dielectric constants of the identified silicone rubber ranges from 1.8–5.2 F/m [16,46,47,48,49,50,51,52,53]. Since the dielectric constant does not change much with frequency, the value for the dielectric constant at a lower frequency remains almost the same as at a higher frequency [16,46,48,49]. For example, the dielectric constant of a pure addition-cured silicone rubber, whose value was 2.8 F/m at 1 kHz remains the same at 1 MHz [46]. Compared to the value of the available commercial silicone rubber, our dielectric constant values for both the platinum-cured silicone (2.81–3.56 F/m) and the condensation-cured silicone rubber (3.65–3.70 F/m) mixed with Slacker additive and/or Thi-vex were mostly in the range of expected values.

The dielectric loss (tan δ) or dielectric loss factor (ε”) are the properties commonly measured for calculating conductivity of a material. The dielectric loss or dielectric loss factor values from the literature were converted into conductivity by using the same equation used in Section 2.1 to compare the conductivity values measured in this study. In contrast to the dielectric constant, dielectric loss or dielectric loss factor depends on the measured frequency [16,46,48,49]. Therefore, the conductivity of a material changes over the measured frequency. According to the technical documents of various commercial silicone rubbers, the typical values of conductivity are in the range of 7.7 × 10^−6^–3.8 × 10^−5^ S/m at 50–100 Hz [21,43,44,45]. Sui et al. and Risse et al. demonstrated that the conductivity increases linearly in a 0.1 Hz–10 MHz frequency range [50,53]. According to a number of literature reports, the dielectric loss of silicone rubber ranges between 1.0 × 10^−12^–1.6 × 10^−5^ S/m, 1.0 × 10^−9^–2.0 × 10^−6^ S/m, 1.0 × 10^−9^–4.4 × 10^−5^ S/m, and 1.1 × 10^−5^–0.00038 S/m at 1 Hz, 100 Hz, 1 kHz, and 1 MHz, respectively [16,46,47,48,49,50,51,52,53]. Most of the dielectric losses and loss factors of the identified silicone rubbers were measured under 10 MHz. Since the dielectric loss of commercial silicone rubber has not been measured at such high frequency (127 MHz) and the value of dielectric loss depends on the measured frequency, a direct comparison with the same material could not be made. Considering that the conductivity increases with frequency and the maximum conductivity values of one of the pure commercial silicone rubber was 0.000382 S/m at 1 MHz [46], the conductivity values of the platinum-cured silicone rubber (0.000154–0.000622 S/m) and the condensation-cured silicone rubber (0.000263–0.000347 S/m) from the results of this study are reasonable.

The compressive modulus of various identified pure commercial silicone rubbers ranged from 200 kPa to 5.9 MPa [49,53,54]. The shear modulus of various identified pure commercial silicone rubbers ranged from 68.9 kPa to 1.83 GPa [54,55]. When comparing with the literature values, the compressive modulus of both pure platinum-cured silicone rubber (1045 kPa) and the pure condensation-cured silicone rubber (489 kPa), as well as the shear modulus of the pure condensation-cured silicone rubber, were in the range of the expected values. However, the shear modulus of the platinum-cured silicone rubber (64 kPa) was found to be slightly lower than the minimum shear modulus value of the identified commercial silicone rubbers.

## 5. Conclusions

Researchers involved in medical device or implant development must consider a wide array of possibilities when fashioning these products. Not only must they be chemically available, but also appropriate for the physiological functionalities of the human body. The safety, efficacy, and longevity of the product is a major concern, as well as the physical characteristics of the material, such as the fabrication methods and elastomer make-up, all of which affect the device manufacturing process [56]. Silicone, in particular, has a widespread range of uses in medical applications due to its specific properties. Its malleability, wide range in temperature stabilities, and potential polymer concoctions allow for novel research in this area. Current common applications of silicone materials include catheters, tubing for feeding, drainage, peristaltic pumps, ear plugs, and shunts, as well as prosthetic devices [57].

This study uncovered relationships between silicone’s dielectric properties and two silicone rubber additives, Thi-vex solution and Slacker additive. While the Slacker additive resulted in an increase in both dielectric constant and conductivity measured, Thi-vex resulted in the decreased dielectric constant and conductivity of silicone rubber. All platinum-cured silicone:Slacker ratios showed exponential decay relationships with % strain in the compression test. Furthermore, a shear test was performed on the platinum-cured silicone up to a 1:0.75 silicone:Slacker ratio. The silicone:Slacker ratios greater than 1:0.75 were unavailable to produce shear moduli data. The compression modulus of the condensation-cured silicone rubber was lower than that of pure platinum-cured silicone rubber, while the shear modulus of the condensation-cured silicone rubber was reco rded as having a higher value than that of the pure platinum-cured silicone rubber.

Our study offers novel information about the dielectric and mechanical properties of two types of silicone rubber, and the changes associated with the addition of two common silicone additives. As highlighted in the literature, the applications of silicone polymers in medical implants and devices are vast and uncharted, and require much research to further our understanding and our applications of these materials. This study offers a quantitative guide to clinicians and engineers in choosing silicone rubbers as a material for their respective projects. The overall impact of our work will allow for the appropriate selection of materials as they pertain to medical implants or bioelectronics, with respect to the dielectric and mechanical properties. Future studies should explore a broader frequency range and higher percentage mechanical strains for data collection and to further knowledge in this area.

## Figures and Tables

**Table 1:** Dielectric properties of the platinum-cured silicone relative to the concentrations of Slacker and Thi-vex.

		Thi-Vex Concentration (%)							
		0.0		0.5		1.0		2.0	
		ε’ (F/m)	σ (S/m)	ε’ (F/m)	σ (S/m)	ε’ (F/m)	σ (S/m)	ε’ (F/m)	σ (S/m)
Silicone:Slacker ratio	1:0.00	3.5329 ± 0.0672	0.000284 ± 0.000021	3.3872 ± 0.3338	0.000381 ± 0.000197	3.5309 ± 0.0633	0.000441 ± 0.000133	3.4346 ± 0.2649	0.000622 ± 0.000033
	1:0.25	3.4443 ± 0.0861	0.000303 ± 0.000050	3.4641 ± 0.0417	0.000322 ± 0.000042	3.4621 ± 0.1472	0.000360 ± 0.000188	3.5533 ± 0.1595	0.000455 ± 0.000318
	1:0.50	3.4346 ± 0.0051	0.000226 ± 0.000009	3.4202 ± 0.0592	0.000264 ± 0.000091	3.4424 ± 0.1290	0.000309 ± 0.000263	3.5621 ± 0.1954	0.000504 ± 0.000358
	1:0.75	3.3788 ± 0.0776	0.000229 ± 0.000135	3.4716 ± 0.0062	0.000318 ± 0.000054	3.4101 ± 0.1122	0.000208 ± 0.000166	3.3851 ± 0.1859	0.000310 ± 0.000275
	1:1.00	3.3147 ± 0.0613	0.000261 ± 0.000051	3.4197 ± 0.0466	0.000302 ± 0.000030	3.3960 ± 0.1110	0.000282 ± 0.000204	3.4019 ± 0.1394	0.000313 ± 0.000240
	1:1.25	3.3101 ± 0.0125	0.000195 ± 0.000005	3.3467 ± 0.0092	0.000305 ± 0.000070	3.3452 ± 0.0941	0.000276 ± 0.000225	3.3871 ± 0.1813	0.000322 ± 0.000353
	1:1.50	3.2293 ± 0.0735	0.000226 ± 0.000056	3.3035 ± 0.2698	0.000307 ± 0.000369	3.3171 ± 0.1752	0.000191 ± 0.000343	3.3341 ± 0.1341	0.000241 ± 0.000230
	1:1.75	3.2195 ± 0.1177	0.000187 ± 0.000135	3.2082 ± 0.1983	0.000233 ± 0.000328	3.2998 ± 0.1418	0.000262 ± 0.000231	3.3225 ± 0.1240	0.000302 ± 0.000247
	1:2.00	2.8075 ± 0.2730	0.00018 ± 0.000255	3.1858 ± 0.2625	0.000154 ± 0.000288	3.2688 ± 0.1410	0.000175 ± 0.000187	3.2468 ± 0.0649	0.000247 ± 0.000174

**Table 2:** Dielectric properties of the condensation-cured silicone relative to the concentrations of Thi-vex.

	Thi-Vex Concentration (%)			
	0.0%	0.5%	1.0%	2.0%
ε’ (F/m)	3.6488 ± 0.0091	3.6608 ± 0.0206	3.6677 ± 0.0144	3.7024 ± 0.0200
σ (S/m)	0.000267 ± 0.000025	0.000263 ± 0.000038	0.000311 ± 0.000046	0.000347 ± 0.000052

**Table 3:** Compressive moduli of the platinum-cured and condensation-cured silicone relative to % strain and Slacker concentrations.

Sample	Slacker Ratio	Modulus at 2% Strain (kPa)	Modulus at 4% Strain (kPa)	Modulus at 6% Strain (kPa)	Modulus at 8% Strain (kPa)	Modulus at 10% Strain (kPa)
Platinum-cured Silicone	1:0.00	433 ± 37	553 ± 31	768 ± 33	953 ± 36	1045 ± 34
	1:0.25	207 ± 13	240 ± 14	300 ± 16	347 ± 18	368 ± 19
	1:0.50	96 ± 6	111 ± 7	137 ± 8	157 ± 7	166 ± 6
	1.0.75	48 ± 2	53 ± 1	60 ± 1	64 ± 1	66 ± 1
	1:1.00	16 ± 1	17 ± 1	18 ± 1	20 ± 1	21 ± 1
	1:1.25	14 ± 1	14 ± 1	15 ± 1	16 ± 1	17 ± 2
Condensation-cured Silicone	N/A	220 ± 15	261 ± 15	349 ± 18	442 ± 20	489 ± 21

**Table 4:** Shear moduli of the platinum-cured and condensation-cured silicone relative to Slacker concentration.

Scheme.	Slacker Ratio	Modulus (kPa)
Platinum-cured Silicone	1:0.00	64 ± 4
	1:0.25	18 ± 1
	1:0.50	3 ± 0.2
	1:0.75	N/A
Condensation-cured Silicone	N/A	87 ± 2

## Data Availability

The data presented in this study are available on request from the corresponding author.

## Appendix A

**Table A1:** Equations and R^2^ values for individual trendlines in Figure 1a.

Thi-Vex (%)	0%	0.5%	1%	2%
Linear equation	y = −0.2704x + 3.5672	y = −0.1288x + 3.4851	y = −0.1234x + 3.5092	y = −0.1265x + 3.5295
R^2^	0.7751	0.7078	0.9805	0.7052

**Table A2:** Equations and R^2^ values for individual trendlines in Figure 1b.

Silicone:Slacker Ratio	Linear Equation	R^2^
1:0.00	y = −0.0257x + 3.4939	0.0917
1:0.25	y = 0.0537x + 3.434	0.8736
1:0.50	y = 0.0685x + 3.4049	0.7971
1:0.75	y = −0.0109x + 3.4209	0.0485
1:1.00	y = 0.0315x + 3.3555	0.3318
1:1.25	y = 0.0353x + 3.3163	0.918
1:1.50	y = 0.0462x + 3.2556	0.7287
1:1.75	y = 0.0595x + 3.2104	0.7912
1:2.00	y = 0.1875x + 2.9632	0.549

**Table A3:** Equations and R^2^ values for individual trendlines in Figure 1c.

Thi-Vex (%)	0%	0.5%	1%	2%
Linear equation	y = −6 × 10^−5^x + 0.0003	y = −7 × 10^−5^x + 0.0004	y = −0.0001x + 0.0004	y = −0.0002x + 0.0005
R^2^	0.669	0.609	0.6759	0.7594

**Table A4:** Equations and R^2^ values for individual trendlines in Figure 1d.

Silicone:Slacker Ratio	Linear Equation	R^2^
1:0.00	y = 0.0002x + 0.0003	0.9953
1:0.25	y = 8 × 10^−5^x + 0.0003	0.974
1:0.50	y = 0.0001x + 0.0002	0.9525
1:0.75	y = −3 × 10^−6^x + 0.0003	0.0024
1:1.00	y = 2 × 10^−5^x + 0.0003	0.6032
1:1.25	y = 5 × 10^−5^x + 0.0002	0.5959
1:1.50	y = −8 × 10^−6^x + 0.0002	0.0206
1:1.75	y = 6 × 10^−5^x + 0.0002	0.958
1:2.00	y = 4 × 10^−5^x + 0.0002	0.6628

**Table A5:** Significant difference in conductivity between various Thi-vex concentrated groups. A significant difference was indicated with O and an insignificant difference was indicated with x.

σ	0%	0.5%	1.0%	2.0%
0%	x	x	x	O
0.5%	x	x	x	x
1.0%	x	x	x	x
2.0%	O	x	x	x

**Table A6:** Significant difference in dielectric constant between various Slacker concentrated groups. A significant difference was indicated with O and an insignificant difference was indicated with x.

ɛ’	1:0.00	1:0.25	1:0.50	1:0.75	1:1.00	1:1.25	1:1.50	1:1.75	1:2.00
1:0.00	x	x	x	x	x	x	x	O	O
1:0.25	x	x	x	x	x	x	x	O	O
1:0.50	x	x	x	x	x	x	x	x	x
1:0.75	x	x	x	x	x	x	x	x	O
1:1.00	x	x	x	x	x	x	x	x	O
1:1.25	x	x	x	x	x	x	x	x	O
1:1.50	x	x	x	x	x	x	x	x	x
1:1.75	O	O	x	x	x	x	x	x	x
1:2.00	O	O	x	O	O	O	x	x	x

**Table A7:** Significant difference in conductivity between various Slacker concentrated groups. A significant difference was indicated with O and an insignificant difference was indicated with x.

σ	1:0.00	1:0.25	1:0.50	1:0.75	1:1.00	1:1.25	1:1.50	1:1.75	1:2.00
1:0.00	x	x	x	x	x	x	x	O	O
1:0.25	x	x	x	x	x	x	x	x	x
1:0.50	x	x	x	x	x	x	x	x	x
1:0.75	x	x	x	x	x	x	x	x	x
1:1.00	x	x	x	x	x	x	x	x	x
1:1.25	x	x	x	x	x	x	x	x	x
1:1.50	x	x	x	x	x	x	x	x	x
1:1.75	O	x	x	x	x	x	x	x	x
1:2.00	O	x	x	x	x	x	x	x	x

**Table A8:** Equations and R^2^ values for individual curves in Figure 3.

% Strain	2%	4%	6%	8%	10%
Curve equation	y = 416.51 × 10^−2.916x^	y = 519.49 × 10^−3.081x^	y = 701.84 × 10^−3.287x^	y = 850.18 × 10^−3.404x^	y = 920.67 × 10^−3.451x^
R^2^	0.9995	0.9989	0.9975	0.996	0.9951

**Table A9:** Equations and R^2^ values for individual trendlines in Figure 4.

Silicone:Slacker Ratio	Linear Equation	R^2^
1:0.00	y = 81.126x + 263.84	0.9845
1:0.25	y = 21.398x + 163.88	0.9795
1:0.50	y = 9.1793x + 78.212	0.98
1:0.75	y = 2.3659x + 43.869	0.968
1:1.00	y = 0.6378x + 14.445	0.9902
1:1.25	0.3642x + 13.271	0.9683
